# Supplementary material for: Factors affecting the maximum outcome payments of social impact bonds
Source: PLoS One. 2025 Jul 7;20(7):e0327547. doi: 10.1371/journal.pone.0327547 (PMC12233227; doi:10.1371/journal.pone.0327547)
Supplement: S1 Fig — (DOC) [file pone.0327547.s001.doc]

**S1 Fig. Research hypothesis**

**Hypothesis 1 (H1). There is a positive correlation between treasury rate and maximum outcome payment.**

**Hypothesis 2 (H2). There is a positive correlation between inflation rate and maximum outcome payment.**

**Hypothesis 3 (H3). There is a positive correlation between bond period and maximum outcome payment.**

**Hypothesis 4 (H4). There is a positive correlation between capital raised and maximum outcome payment.**

**Hypothesis 5 (H5). There is a negative correlation between size of the target population and maximum outcome payment.**
