# Supplementary material for: Factors affecting the maximum outcome payments of social impact bonds
Source: PLoS One. 2025 Jul 7;20(7):e0327547. doi: 10.1371/journal.pone.0327547 (PMC12233227; doi:10.1371/journal.pone.0327547)
Supplement: S2 Fig — (DOC) [file pone.0327547.s002.doc]

**S2 Fig. Regression coefficients plot**

The regression coefficient plot supports the conclusions of the regression analysis: TR and BP have no significant effect on MP, whereas CPI, CR, and TP have a significant impact on MP.
